# Glyphosate exposure in pregnancy and shortened gestational length: a prospective Indiana birth cohort study

**DOI:** 10.1186/s12940-018-0367-0

**Published:** 2018-03-09

**Authors:** S. Parvez, R. R. Gerona, C. Proctor, M. Friesen, J. L. Ashby, J. L. Reiter, Z. Lui, P. D. Winchester

**Affiliations:** 1 Department of Environmental Health Science, Indiana University Fairbanks School of Public Health, 1050 Wishard Boulevard, Indianapolis, IN 46202 USA; 2 Department of Gynecology, Obstetrics and Reproductive Sciences, University of California San Francisco, 505 Parnassus Ave Moffitt Hospital M879B, San Francisco, CA 94143 USA; 3 Franciscan Health, 8111 S Emerson Avenue, Indianapolis, IN 46237 USA; 4 Neonatal-Perinatal Medicine, Riley Children’s Hospital, Indiana University School of Medicine, 699 Riley Hospital Dr RR 208, Indianapolis, IN 46202 USA; 5 Department of Obstetrics and Gynecology, Indiana University School of Medicine, 1044 W. Walnut, R4 035, Indianapolis, IN 46202 USA; 6 Department of Biostatistics, Indiana University Fairbanks School of Public Health, 410 W. Tenth St., Suite 3000, Indianapolis, IN 46202 USA

**Keywords:** Glyphosate, Roundup, Herbicides, Pregnancy, Exposure assessment, Gestational length, Birth weight percentile, Head circumference, Birth outcomes, Caffeine

## Abstract

**Background:**

Glyphosate (GLY) is the most heavily used herbicide worldwide but the extent of exposure in human pregnancy remains unknown. Its residues are found in the environment, major crops, and food items that humans, including pregnant women, consume daily. Since GLY exposure in pregnancy may also increase fetal exposure risk, we designed a birth-cohort study to determine exposure frequency, potential exposure pathways, and associations with fetal growth indicators and pregnancy length.

**Method:**

Urine and residential drinking water samples were obtained from 71 women with singleton pregnancies living in Central Indiana while they received routine prenatal care. GLY measurements were performed using liquid chromatography-tandem mass spectrometry. Demographic and survey information relating to food and water consumption, stress, and residence were obtained by questionnaire. Maternal risk factors and neonatal outcomes were abstracted from medical records. Correlation analyses were used to assess relationships of urine GLY levels with fetal growth indicators and gestational length.

**Results:**

The mean age of participants was 29 years, and the majority were Caucasian. Ninety three percent of the pregnant women had GLY levels above the limit of detection (0.1 ng/mL). Mean urinary GLY was 3.40 ng/mL (range 0.5–7.20 ng/mL). Higher GLY levels were found in women who lived in rural areas (*p* = 0.02), and in those who consumed > 24 oz. of caffeinated beverages per day (*p* = 0.004). None of the drinking water samples had detectable GLY levels. We observed no correlations with fetal growth indicators such as birth weight percentile and head circumference. However, higher GLY urine levels were significantly correlated with shortened gestational lengths (r = − 0.28, *p* = 0.02).

**Conclusions:**

This is the first study of GLY exposure in US pregnant women using urine specimens as a direct measure of exposure. We found that > 90% of pregnant women had detectable GLY levels and that these levels correlated significantly with shortened pregnancy lengths. Although our study cohort was small and regional and had limited racial/ethnic diversity, it provides direct evidence of maternal GLY exposure and a significant correlation with shortened pregnancy. Further investigations in a more geographically and racially diverse cohort would be necessary before these findings could be generalized.

## Background

Glyphosate (GLY, N-phosphonomethylglycine) is a broad-spectrum phosphate herbicide. GLY is the active ingredient in the herbicide Roundup, which is the most heavily used herbicide worldwide [1–4]. In the United States alone, nearly 300 million pounds are applied each year [5]. GLY usage is heaviest in the Midwest due to corn and soybean production. Agricultural run-off has resulted in high detection rates of GLY in US streams, rivers, and lakes [6–8]. In addition, crops that are genetically modified to be GLY resistant (i.e., Roundup-ready) have GLY residue. Over 90% of corn, soy, and canola grown in the United States are modified in this way, and these grains are used in most processed foods [2].

In utero exposure to either Roundup or GLY alone has been linked to birth defects and fetal loss in chickens, frogs, and mammals [9–11]. While these studies did not replicate human exposure in the low dose range, they found fetal toxicity risks such as dilated hearts and visceral anomalies in rats and post-implantation loss and late embryonic deaths in rabbits at doses as low as 20 mg/kg/day. GLY also has been shown to disrupt important pathways in development, such as retinoic acid signaling and estrogen biosynthesis [11]. Further, GLY has been reported to disrupt enzymatic pathways, such as the cytochrome P450, and to damage DNA structure in human breast epithelial and placental cells [12–14]. GLY inhibits aromatase (CYP19A1) activity by a direct interaction with the active site of the enzyme at concentrations 100 times lower (0.036 g/L) than the recommended use in agriculture, i.e., 3.6 g/L [12]. Moreover, GLY induced DNA damage and chromosomal breaks in vitro and in vivo in mice [15].

Despite evidence of potential genotoxicity and teratogenic activity of GLY in animal studies, GLY effects on human pregnancy and fetal development have not been investigated. Epidemiological evidence of GLY exposure effects on reproductive and developmental health outcomes is limited. A systematic review conducted in 2016 found only ten studies that had tested the association between indirect measure of GLY exposure and adverse pregnancy outcomes [16]. A California-based study on a rural pregnant population for their residential proximity to farmland and exposure during the pre- and post-conception period (i.e., 4 weeks before and 8 weeks after conception) reported an increased risk of neural tube defects (Odds ratio (OR) = 1.5; 95% confidence interval (CI), 1.0-2.4) [17, 18]. A Minnesota Red River Valley study reported a significant association between male perinatal exposure to multiple pesticides including GLY with increased risk (OR = 3.6; 95% CI, 1.0-4.0) of attention deficit hyperactivity disorder [19, 20]. In addition, a few reproductive epidemiological studies have evaluated GLY association with non-congenital anomalies with mostly negative results. These include the Canadian Ontario Farm Family Health Study [21–24], a Columbian Study, and the Iowa and North Carolina Farm and Nonfarm Family Studies [25, 26]. Only the Ontario Farm Family Health study reported significant association between perinatal exposure to GLY (and other pesticides) and increased risk (OR = 1.7; 95% CI, 1.0-1.6) of spontaneous abortion later in pregnancy (12–19 weeks). Thus, most prior epidemiological studies have been of limited sizes and findings have been inconclusive due to methodological limitations, lack of direct measurement of GLY, and without definitive evidence that GLY exposure harms human fetal development [17–26]. Despite these inconclusive epidemiological studies, the prevalence of GLY residues in genetically modified crops and contaminated drinking water [1–6, 8, 27] warrants further investigation to determine the risk of adverse fetal outcomes due to GLY exposure.

North American and European studies that measured GLY in the urine or serum of individuals reported GLY levels varied by geographical area and occupation type [28–32]. Some studies have used an immunoassay methodology to measure GLY levels, which is considered a less reliable method due to lower sensitivity and higher false positive rates; the limit of detection (LOD) for immunoassay ranged from 0.9 to 7.5 ng/mL [28, 31]. Other studies that used more sensitive gas chromatography-mass spectrophotometery (GC-MS) or high performance liquid chromatography-mass spectrophotometery (HPLC/MS) techniques reported LOD of 0.15 ng/mL or 2 ng/mL, respectively [29, 30, 33, 34]. However, these studies did not assess the association between GLY exposure and adverse pregnancy outcomes.

Current evidence for an association between GLY exposure and elevated risk of adverse reproductive and developmental outcomes is limited and inconsistent. Studies that relied on indirect estimates of GLY exposure to investigate risks of congenital birth defects or other developmental outcomes could not reliably estimate the timing or dose of exposure during pregnancy. GLY exposure was estimated from direct pesticide use (pesticide user vs. non-user during pregnancy), residential proximity to areas with pesticide use, and multiple pesticide use as a proxy for GLY exposure [35].

Despite, the inconclusive epidemiological evidence, the risk of exposure is predicted to be high due to the prevalence of GLY residues in genetically modified crops and contaminated drinking water [1–6, 8, 27]; thus, further investigation to determine the risk of GLY exposure and adverse fetal outcomes is warranted. To address this need, we designed a prospective birth-cohort study to test the hypothesis that GLY can be directly measured in the urine of most pregnant women due to dietary ingestion of contaminated food, beverages, and drinking water, and that higher GLY levels in pregnancy will correlate with adverse fetal outcomes. The goals of this study were 1) to determine the prevalence and exposure levels of GLY in pregnant women, 2) to assess the contribution of demographic factors, diet, and drinking water as potential exposure risks, and 3) to evaluate the association between urine GLY level with pregnancy length and fetal growth.

## Methods

### Recruitment of participants

This study was approved by the Indiana University Institutional Review Board (#1504233705). The participants were recruited following referral from their obstetrician at a private obstetrical practice in Central Indiana from June 2015 to June 2016. Enrollment included 77 pregnant women between the ages of 18 and 40 years during their routine obstetrical prenatal visit. Signed informed consent and HIPAA authorizations were obtained from all participants. Six participants were excluded from the study: two were ineligible due to higher maternal age, two encountered pregnancy loss during the study period, and two changed obstetrical care providers. A subset of (*n* = 71) pregnant women with a singleton live birth were measured for urine GLY. The subject selection was random within each drinking water source (e.g., public water supply and private well water). All participants received monetary compensation for their participation in the study.

### Study questionnaire

Each participant was asked to complete a questionnaire, which was administered online using REDCap or in a paper form at a subsequent prenatal clinical visit. The questionnaire included dietary and drinking water intake, occupation, and demographic characteristics. The primary variables were: race/ethnicity, maternal age, maternal education, state of residence, county of residence, zip code of residence, marital status, occupation, trimester of sample collection, drinking water source, frequency and volume of water consumption, frequency of organic food intake, recent application of GLY, stress levels, caffeine consumption, smoking status, and alcohol consumption.

### Abstraction of medical records

The participants’ medical charts were reviewed following delivery of their babies. Each maternal record was reviewed for pregnancy risk factors, gestational length, and fetal growth indicators, i.e., birth weight percentile and head circumference. Gestational length was calculated based on last menstrual period and obstetrical adjustment by first ultrasound. Maternal records included: pre-pregnancy characteristics, complications during pregnancy, pregnancy length, and newborn characteristics. *Prepregnancy factors included:* maternal age, pre-existing diseases (i.e., diabetes, hypertension), insurance, substance use, parity, plurality, and prior adverse pregnancy outcomes. *Pregnancy factors included:* prenatal care, singleton gestational, and weight gain during pregnancy, hypertension, diabetes, medications, substance and/or tobacco use, duration of pregnancy, complications of labor and delivery, and method of delivery. *Neonatal factors included:* gender and fetal growth indicators (birth weight, gestational adjusted birth weight percentile, and head circumference). Any abnormal course in post-delivery of the newborn such as congenital anomalies, higher level of care, longer length of stay, and health conditions at discharge were also recorded.

### Specimen collection

Urine specimens were obtained as residual samples from the pregnant women that were collected as part of routine prenatal care. A urine sample was collected from each woman at the initial time of enrollment and at a subsequent clinical visit between 11 and 38 weeks of gestation. Upon enrollment, each participant was provided with a drinking water collection kit and written and verbal instructions for consistency in drinking water collection and to avoid contamination. A residential drinking water sample was collected the same day as the second prenatal urine sample. On the day of urine collection, all study samples were transported to the study’s hospital laboratory for processing. All study samples were de-identified, stored at -70 °C, and then shipped to the University of California San Francisco Clinical Toxicology and Environmental Biomonitoring Laboratory for GLY analysis. Although we had two urine samples from each pregnant woman, GLY measurements were obtained from their second urine sample since it was collected on the same day as their water sample, and both urine and water were analyzed for GLY.

### Analytical method

GLY measurements in urine and drinking water were obtained by liquid chromatography-tandem mass spectrometry (LC-MS/MS) using an Agilent LC 1260 system (Agilent LC 1260- AB Sciex 5500 Triple Quadrupole MS, St. Clara, CA) with an Obelisc-N mixed-mode column (2.1x100mm 5 μm) that was maintained at 40 °C. A 25 μL sample was injected into the column and GLY was eluted using a mobile phase of 1% formic acid in bisphenol-A free water at a flow rate of 1 mL/min. The total run time was 6 min. External calibration was used for drinking water analysis and standard addition for urine analysis. Mass spectral analysis was performed using an electrospray ionization source operated in negative mode. The parameters used for ionization include curtain gas 20 psi, collision gas 9 psi, ion spray voltage -4500 V, temperature 700 °C, and ion source gas 60 psi. GLY was monitored using two transitions: 168.1–62.9 m/*z* (qt) and 168.1–81.0 m*/z* (ql). We used 2-^13^C-*N*-GLY as an internal standard and monitored using the 169.4–63.0 m*/z* transition. Quantitative analysis of GLY was done by isotope dilution method. All measured urine samples were corrected for specific gravity in our analysis for differences in urinary dilution. The established limits of quantification (LOQ) and LOD for GLY in drinking water were 0.08 and 0.02 ng/mL, respectively, while the LOQ and LOD in urine were 0.5 and 0.1 ng/mL, respectively. These limits are lower than the two previously reported methods on urine GLY measurement [36, 37].

### Statistical analyses

The primary measures, urine GLY, indicators of fetal growth (e.g., birthweight percentile, head circumference), and gestational length, were continuous numerical variables. Continuous numerical variables were assessed for central tendency, dispersion, and skewness before formal analyses. GLY levels were summarized as quartiles, mean, minimum, and maximum. In five cases where GLY was less than the LOD, 0.1 ng/mL was substituted as the measured level for subsequent statistical analysis. Since continuous variables exhibited deviations from normality (i.e., non-parametric data) upon preliminary examination, Kruskal Wallis, a rank-based nonparametric test, was used for group comparisons. The rank-based Kruskal Wallis test provides a robust non-parametric alternative to traditional linear regression which requires certain underlying conditions or assumptions to be met, particularly for smaller sample sizes [38, 39]. Questionnaire-based categorical variables were summarized as groups (sub-categories) to compare mean urine GLY levels using nonparametric chi-square tests or Fisher’s exact tests as appropriate. The Chi-square test was used to determine whether there was a significant difference across different groups for urine GLY levels, while Fisher’s exact test was used to determine whether there was a significant difference between any two groups for urine GLY levels [40]. Nonparametric Spearman partial correlations were developed to quantify associations between two continuous variables while controlling for potential confounders. This includes association between the independent variable, urine GLY levels, and a dependent variable (e.g., birth weight percentile, head circumference, and gestation length) adjusted for confounders. The key confounders included in correlation analysis were maternal age, pre-pregnancy body mass index, tobacco use, alcohol use, and trimester of pregnancy. All analyses were performed using R package (R 3.3.3, R Core Team). Two-sided *p*-values < 0.05 were considered statistically significant.

## Results

A total of 71 pregnant women with live-born singleton infants were included in this study; the dropout rate was less than 1%. Mean maternal age was 29 years (range 18–39 years). Maternal race was Caucasian (94.2%) and Asian (5.8%). The study population included participants from nine counties in Central Indiana which represented a mix of rural, suburban, and urban addresses, as well as, public and well water supplies.

### Maternal urine GLY levels

The maternal population was used to determine the distribution of measured GLY in urine (Table 1). GLY levels were estimated as quartiles, mean, standard deviation (SD), coefficient of variance (CV), minimum, and maximum. GLY levels above the LOD > 0.1 ng/mL were found in 66 of 71 (93%) urine samples. The mean (±SD) and median GLY levels were 3.40 ± 1.24 and 3.25, respectively. The minimum and maximum concentrations of urinary GLY were 0.5 ng/mL and 7.20 ng/mL, respectively. The CV was 0.36, which indicated low exposure variability across participants in the study. The estimated mean GLY concentrations with and without the five urine samples that were below LOD were not statistically significantly different (*p* = 0.43). Therefore, urine GLY levels for all 71 participants were used for the correlation analysis.

**Table 1:** Summary of the urine GLY levels (ng/mL) and maternal characteristics in the Indiana birth cohort

Covariate	N (%)	Q1	Q2	Q3	Q4	IQR	Mean	SD	Min	Max	CV	*p*-value
Glyphosate (ng/mL)	71 (100)	2.84	3.25	3.91	7.20	1.06	3.40	1.24	0.50	7.20	0.36	
Ethnicity/Race												0.17
ᅟNon-Hispanic White	67 (94.4)	2.84	3.23	3.81	7.20	0.97	3.36	1.25	0.50	7.20	0.37	
ᅟAsian	4 (5.6)	3.55	4.28	4.82	4.89	1.27	4.10	0.93	2.93	4.89	0.23	
Infant Sex												0.79
ᅟMale	38 (53.5)	2.86	3.21	4.30	7.20	1.44	3.52	1.27	0.50	7.20	0.36	
ᅟFemale	33 (46.5)	2.84	3.30	3.74	6.69	0.90	3.26	1.22	0.50	6.69	0.37	
Maternal Education												0.09
ᅟHigh school diploma or less	14 (19.7)	3.18	3.70	4.79	5.69	1.61	3.83	1.34	0.50	5.69	0.35	
ᅟAssociate’s or some college	23 (32.4)	2.73	3.05	3.97	7.20	1.23	3.24	1.38	0.50	7.20	0.42	
ᅟBachelor’s degree or higher	34 (47.9)	2.84	3.26	3.59	6.69	0.75	3.33	1.10	0.50	6.69	0.33	
Residential Area												0.01
ᅟRural	14 (19.7)	3.33	4.05	5.24	7.20	1.90	4.19	1.58	0.50	7.20	0.38	
ᅟSuburban	50 (70.4)	2.76	3.05	3.58	6.69	0.82	3.17	1.13	0.50	6.69	0.36	
ᅟUrban	7 (9.9)	3.19	3.41	3.74	4.24	0.55	3.47	0.50	2.77	4.24	0.14	
Organic Food Consumption												0.62
ᅟNever	10 (14.1)	2.86	3.67	4.75	7.20	1.89	3.86	1.83	0.50	7.20	0.47	
ᅟRarely	39 (54.9)	2.84	3.20	3.81	6.69	0.98	3.37	1.24	0.50	6.69	0.37	
ᅟAlways or frequently	22 (31.0)	2.90	3.32	3.63	4.80	0.73	3.25	0.91	0.50	4.80	0.28	
Corn/Soy Consumption												0.69
ᅟ2 days per week or less	5 (7.0)	3.23	3.26	3.75	4.53	0.52	3.50	0.67	2.75	4.53	0.19	
ᅟ3 days per week or more	66 (93.0)	2.84	3.24	3.92	7.20	1.08	3.39	1.28	0.50	7.20	0.38	
Drinking Water Source												0.11
ᅟPublic water (some bottled water)	24 (33.8)	3.04	3.41	4.32	7.20	1.28	3.74	1.25	0.50	7.20	0.33	
ᅟPublic supply	42 (59.2)	2.75	3.16	3.59	6.69	0.84	3.19	1.25	0.50	6.69	0.39	
ᅟPrivate well	4 (5.6)	2.81	2.98	3.55	4.85	0.74	3.38	0.99	2.72	4.85	0.29	
ᅟNot reported	1 (1.4)	4.23	4.23	4.23	4.23	0.00	4.23	NA	4.23	4.23		
Pre-pregnancy BMI												0.15
ᅟLess than 25	33 (46.5)	2.94	3.23	4.80	7.20	1.86	3.59	1.53	0.50	7.20	0.43	
ᅟ25 to 30	20 (28.2)	2.82	3.59	4.23	4.85	1.41	3.54	0.76	1.97	4.85	0.21	
ᅟGreater than 30	18 (25.4)	2.75	3.05	3.30	4.27	0.56	2.89	0.96	0.50	4.27	0.33	
Stressful Pregnancy												0.21
ᅟNot stressful	27 (38.0)	2.78	3.13	3.65	5.69	0.87	3.29	0.95	0.50	5.69	0.29	
ᅟSomewhat stressful	37 (52.1)	2.94	3.30	3.74	7.20	0.80	3.37	1.26	0.50	7.20	0.37	
ᅟVery stressful	6 (8.5)	3.38	4.61	5.39	6.69	2.01	4.17	2.17	0.50	6.69	0.52	
ᅟNot reported	1 (1.4)	2.75	2.75	2.75	2.75	0.00	2.75	NA	2.75	2.75		
Consume Caffeine												0.99
ᅟNo	9 (12.7)	2.80	3.30	3.75	4.89	0.95	3.49	0.84	2.66	4.89	0.24	
ᅟYes	62 (87.3)	2.85	3.24	3.92	7.20	1.07	3.39	1.30	0.50	7.20	0.38	
Caffeine Intake												0.02
ᅟLess than 8 oz	16 (22.5)	2.76	3.55	4.32	7.20	1.57	3.52	1.45	0.50	7.20	0.41	
ᅟ8 oz. to 24 oz	38 (53.5)	2.85	3.18	3.54	5.34	0.69	3.05	1.04	0.50	5.34	0.34	
ᅟ24 oz. or more	8 (11.3)	3.67	4.91	5.60	6.69	1.92	4.69	1.34	2.84	6.69	0.29	
ᅟNot reported	9 (12.7)	2.80	3.30	3.75	4.89	0.95	3.49	0.84	2.66	4.89	0.24	
Household Income												0.13
ᅟLess than $50,000	22 (31.0)	3.07	3.33	3.84	4.93	0.78	3.40	0.87	0.50	4.36	0.26	
ᅟ$50,000 to $99,999	27 (38.0)	2.71	2.93	3.53	6.69	0.82	2.96	1.40	0.50	6.69	0.47	
ᅟ$100,000 or more	15 (21.1)	2.96	3.23	3.66	4.89	0.71	3.44	0.70	2.66	4.89	0.20	
ᅟNot reported	7 (9.9)	4.09	5.34	5.63	7.20	1.54	5.01	1.37	3.13	7.20	0.27	
Maternal Age												0.46
ᅟLess than 20	2 (2.8)	3.24	3.45	3.66	3.87	0.42	3.45	0.60	3.03	3.87	0.17	
ᅟ20 to 35	64 (90.1)	2.82	3.21	3.86	7.20	1.04	3.37	1.30	0.50	7.20	0.38	
ᅟGreater than 35	5 (7.0)	3.30	3.41	3.94	4.80	0.63	3.75	0.64	3.30	4.80	0.17	
Hypertension												0.28
ᅟNo	52 (73.2)	2.83	3.21	3.75	7.20	0.92	3.33	1.27	0.50	7.20	0.38	
ᅟYes	19 (26.8)	2.96	3.52	4.10	5.69	1.15	3.59	1.17	0.50	5.69	0.33	
Diabetes												0.08
ᅟNo	65 (91.5)	2.83	3.20	3.74	7.20	0.91	3.36	1.29	0.50	7.20	0.38	
ᅟYes	6 (8.5)	3.49	3.84	4.19	4.53	0.69	3.85	0.50	3.23	4.53	0.13	
Drug Use												NR
ᅟNo	69 (97.2)	2.84	3.25	3.87	7.20	1.03	3.42	1.20	0.50	7.20	0.35	
ᅟYes	1 (1.4)	4.93	4.93	4.93	4.93	0.00	4.33	NA	4.93	4.93		
ᅟNot reported	1 (1.4)	0.50	0.50	0.50	0.50	0.00	0.50	NA	0.50	0.50		
Tobacco Use												0.93
ᅟNo	59 (83.1)	2.91	3.26	3.75	7.20	0.84	3.42	1.14	0.50	7.20	0.33	
ᅟYes	12 (16.9)	2.62	3.12	4.63	5.69	2.01	3.30	1.73	0.50	5.69	0.52	
Alcohol Use												0.86
ᅟNo	68 (95.8)	2.84	3.26	3.89	7.20	1.05	3.39	1.25	0.50	7.20	0.37	
ᅟYes	3 (4.2)	2.88	3.05	3.99	4.93	1.11	3.56	1.20	2.70	4.33	0.34	

*Legend:* N = number of samples; Q1 = first quartile; Q2 = second quartile; Q3 = third quartile; Q4 = fourth quartile; IQR = inter-quartile range; Mean = mean GLY value of each group; Min = minimum value in the group; SD = standard deviation of group; CV = coefficient of variation; p-value derived from Kruskal Wallis chi-square test at significance level of 0.05 for comparisons across groups; NR = not reported due to one case of drug use; *NA* = not applicable

#### (a) Relationship between urine GLY levels and survey variables

The analysis of measured urinary GLY levels in the pregnant women was stratified based on the administered survey variables on demographic determinants, dietary and drinking water intake, and infant sex (Table 1). Based on the survey responses, few mothers smoked (16.9%), consumed alcohol (4.2%), or used drugs (1.4%) during pregnancy. The majority of participants had body mass index < 30 (74.6%), a college degree (47.9%), a household income of $50,000 or more per annum (69%), lived in suburban and urban areas (80.3%), ate processed food (69%), drank water from a public water system (93%), and consumed caffeine (87.3%).

**Fig. 1:**
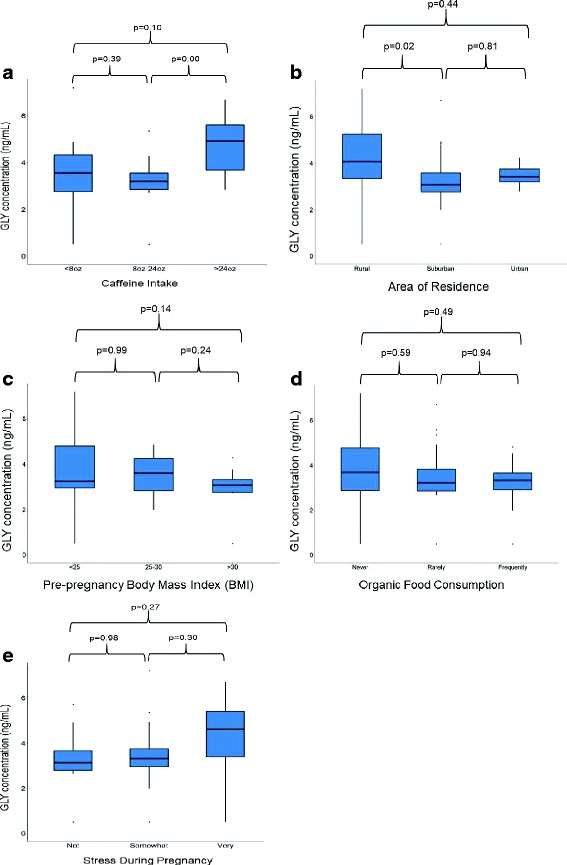
Trends in urine GLY levels with key maternal characteristics and behavior: (**a**) Caffeine Intake, (**b**) Area of Residence, (**c**) Pre-pregnancy Body Mass Index (BMI), (**d**) Organic Food Consumption, and (**e**) Stress During Pregnancy. *Legend:* For group comparison, the *p* values were calculated using Fisher’s exact tests at significance level of 0.05. (**a**) The number of subjects with caffeine intake: < 8 oz. = 16, 8-24 oz. = 38, and > 24 oz. = 8; (**b**) number of subjects that lived in the residential area: rural = 14, suburban = 50, and urban = 7; (**c**) number of subjects with pre-pregnancy BMI: < 25 = 33, 25–30 = 20, and > 30 = 18; (**d**) number of subjects with frequency of organic food consumption: never = 10, rarely = 29, and frequently = 32; and (**e**) number of subjects with the degree of stress during pregnancy: not = 27, somewhat = 37, and very stressed = 6

Maternal area of residence and caffeine consumption were the only two variables that were significantly associated with urine GLY measures (Table 1). The urine GLY levels were substantially higher for women who lived in rural areas (*p* = 0.02) or consumed > 24 oz. of caffeine (*p* = 0.004) compared to women who lived in urban or suburban areas, or consumed < 24 oz. of caffeine per day (Fig. 1). Though not statistically significant, there were trends towards lower GLY with increasing pre-pregnancy BMI, increasing organic food intake, and lower stress levels reported by participants (Fig. 1). No correlations were found between GLY levels and maternal age (*p* = 0.46), gender of newborn (*p* = 0.79), maternal education (*p* = 0.09), genetically modified vs organic food (*p* = 0.62), drinking water sources (*p* = 0.11), pre-pregnancy BMI (*p* = 0.15), stress during pregnancy (*p* = 0.21), income levels (*p* = 0.13), tobacco use (*p* = 0.93), or alcohol use (*p* = 0.86) [Table 1].

#### (b) Correlation between urine GLY and parameters of fetal growth and gestational length

Two infants were born preterm, i.e., < 37 weeks of gestation. The overall mean (± SD) gestational age of all 71 births was 273.86 ± 7.0 days, mean birth weight was 3412 ± 535 g, and head circumference was 34.4 ± 1.9 cm (Table 2). The mean maternal age was 29 years, and pre-pregnancy BMI was 27. Comparison of overall mean with newborn sex-specific means of maternal age, pre-pregnancy BMI, fetal growth outcomes, and gestational length showed no statistically significant differences. We did not find an effect of BMI on urine GLY levels. Some human studies on similar compounds such as organophosphate pesticides also reported no association with BMI, while others have found inverse or positive correlations [41, 42]. Therefore, there is no conclusive evidence that BMI is associated with urinary excretion of GLY.

**Table 2:** Overall and newborn sex-specific means (+/- SD) of maternal age, pre-pregnancy BMI, and birth outcomes in the Indiana birth cohort

Variables	Overall Mean	Mean for Male	Mean for Female	*p*-value
Maternal age (year)	29.01 ± 4.66	29.03 ± 5.03	29.0 ± 4.26	0.99
Gestation age (days)	273.86 ± 7.15	273.71 ± 8.62	274.03 ± 5.07	0.98
Body weight (g)	3412.45 ± 534.65	3483.39 ± 571.97	3330.76 ± 483.93	0.49
Head circumference (cm)	34.39 ± 1.92	34.75 ± 2.15	33.99 ± 1.56	0.27
Pre-pregnancy BMI	26.95 ± 5.42	26.48 ± 5.48	27.48 ± 5.39	0.74

**Fig. 2:**
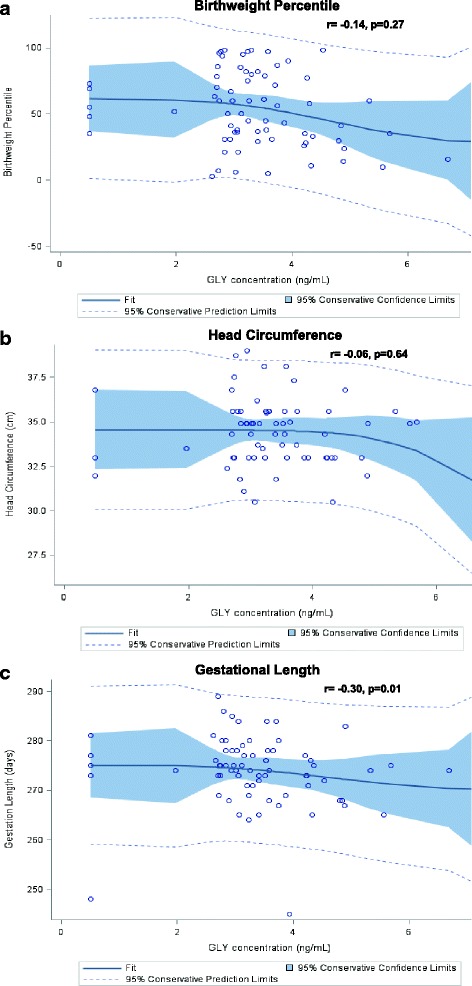
Monotonic relationships between urine GLY in pregnant women and their birth outcomes: (**a**) Birthweight Percentile, (**b**) Head Circumference, and (**c**) Gestational Length. *Legend:* A monotonic relationship shows that variables are changing concurrently, but not at the same rate, thus, this relationship is not linear. Spearman’s rank-based correlations (r) show here the strength and direction of the monotonic relationships between the urine GLY measures and birth outcomes. *p*-values were calculated at significance level of 0.05 with key confounders. Correlation analysis was adjusted for the confounders maternal age, BMI, tobacco use, alcohol use, and trimester of pregnancy

Spearman partial correlation analysis was performed for association between measured GLY level and the indicators of fetal growth and gestational age in the presence of key confounders (Fig. 2). A significant negative correlation was found between urine GLY levels and gestational length (r = − 0.30, *p* = 0.01). However, no correlation was found with reduced birth weight (r = − 0.14, *p* = 0.27) or head circumference r = − 0.06, *p* = 0.64).

## Discussion

The main finding of this study was that a very high proportion (93%) of pregnant women from a Midwest obstetrical practice have detectable levels of GLY in their urine. The high rate of detection in our study was comparable to the detection rates reported in the Iowa study where the frequencies of GLY detection in farm, non-farm, father, mother, and children were reported between 65% and 88% [28]. Our study sampled women more recently (2015–2016) than the Iowa Study (2001), and it is likely that GLY exposure has gone up over time as was reported by Mills et al., 2017 [43]. Another explanation of why the frequency of urine GLY detection in our study was higher than the reported levels in the Minnesota Red River Valley and South American studies is because our LOD was 10 times lower than in these studies (0.1 vs 1 ng/mL) [31, 32]. In a recent German study, GLY was detected in a significant number of individuals who consumed meats. In that study, people who consumed conventional (*n* = 99) vs organic diets (*n* = 41) had significantly higher urine GLY levels (*p* = 0.0002). Also, people who had chronic diseases (*n* = 102) *vs*. healthy subjects (*n* = 199) had significantly higher urine GLY levels (*p* = 0.03). The reported mean urine GLY level was 5.4 ± 11.5 μg/mL [33].

In the Indiana birth cohort, all drinking water samples had no detectable GLY. Thus, it is unlikely that the source of GLY exposure in these women was drinking water. Water treatment processes that use alum as a coagulant to remove turbidity, also remove GLY [44]. Since we did not measure GLY residues in participants’ food and beverages in our study, we relied on participants’ response for frequency of organic food intake and caffeinated beverages to determine correlations with urine GLY levels. However, previous studies have suggested that the likely primary sources of GLY exposure was the diet. For example, the European Food Safety Authority (EFSA) database lists soybeans, corn, barley, lentils, linseed, mustard seed, oats, sorghum, wheat, coffee beans, tea, beet root, and mushroom as crops with GLY residues [45]; Bohn et al. (2014) demonstrated that genetically modified soy beans have significant GLY and metabolite AMPA (α-amino-3-hydroxy-5-methyl-4-isoxazolepropionic acid) residues (GLY = 3.3 mg/kg and AMPA = 5.7 mg/kg) [46]; the Canadian Food Inspection Agency (CFIA) in 2015–2016 reported GLY residues in 29.7% of food samples. The foods with the highest residue rates were beans, peas, lentils, grains, infant cereal and baby food, followed by juice and other processed forms of fruits and vegetables [47]. Recently, the Food and Drug Administration (FDA) began preliminary testing of samples of soybeans, corn, milk, and eggs for glyphosate residues in 2017, which implies that these commodities may have residual GLY [48].

Contrary to previous studies on GLY residues in dietary food items, our study showed no correlation (*p* = 0.62) between urinary GLY levels with increasing frequency of organic food consumption (never = 3.86 ng/mL > rarely = 3.37 ng/mL > frequently = 3.25 ng/mL). However, it is difficult to confirm these findings in our study due to the small size of the cohort and potential for reporting error and recall bias. Similarly, it is difficult to conclude caffeinated beverages contain high GLY residues based on our finding that urine GLY levels were significantly correlated (*p* = 0.004) with the consumption of caffeine containing products (e.g., coffee, tea, and soft drinks). It is possible that some caffeine-containing products have GLY; however, our questionnaire did not differentiate between caffeine products and hence the evidence is only suggestive. Alternatively, our results suggest that high doses of caffeine may alter urine levels of GLY through a diuretic effect [49, 50].

Pregnant women from rural areas had significantly higher urinary GLY levels than suburban residents (*p* = 0.02). Since the majority of rural participants were not farmers or directly involved in Roundup application, this suggests the inhalation of contaminated air or dust may represent another exposure pathway for higher urine GLY levels in rural areas. However, residential air samples were not collected in this study so this cannot be confirmed. While Curwin et al. did not find significant differences in GLY levels between children in farm vs. non-farm families [28], other pesticide studies have reported an association between exposure levels and proximity to agricultural fields [30, 51, 52].

This study is the first to correlate direct measures of GLY exposure in pregnancy with fetal growth indicators and pregnancy length. Despite a small cohort size, we found a small negative (r = − 0.30) but significant correlation between urine GLY levels and gestational length with confounders (*p* = 0.01). Previous studies have also linked pesticide exposure with shorter gestation but neither study specifically measured GLY [53, 54].

This study reinforces a growing body of evidence suggesting that pesticide exposure in pregnancy may be correlated with gestational length, as well as adverse fetal growth. Reduction in gestational length is now known to correlate with life-long adverse consequences. Recent evidence suggests that shortened gestations of one week at term is associated with a reduction in lifetime cognitive achievement [55]. Barker et al., have shown that lower birth weight percentile represents an increased risk for adult metabolic syndrome, hypertension, and coronary death [56]. Our study showed no correlation of reduced birth weight percentile (r = − 0.14, *p* = 0.27) and head circumference (r = − 0.06, *p* = 0.64) with increasing GLY levels. Also, our study did not report any correlations between increasing urine GLY levels and decreasing pre-pregnancy BMI (*p* = 0.15), decreasing organic food consumption (*p* = 0.62), and increasing stress during pregnancy (*p* = 0.2). However, further investigation in a larger cohort is warranted to confirm these findings.

The majority of participants in our cohort were privately insured, Caucasian, non-obese, college educated, had household income above the national average, did not consume alcohol or smoke, and lived in urban or suburban areas. The homogeneity of the cohort made the correlations between glyphosate and adverse pregnancy outcomes less sensitive to potential confounding by those variables related to race and socioeconomic factors.

Although this study provides new information about GLY exposure in pregnancy and birth outcomes, there are several limitations. The maternal cohort size is small, and of limited racial, age, and geographic diversity. Although this lack of diversity likely contributed to the correlations between GLY and gestational length, it limited our ability to generalize these findings to a more diverse population. Many factors (especially race) have significant effects on gestational length and birth weight and additional data from ethnically diverse groups would be required before our findings could be generalized. To determine correlations between GLY levels and other adverse pregnancy outcomes such as birth defects, miscarriage, preterm births, low birth weight, and small for gestational age will require much larger cohort sizes across diverse populations; thus, we did not investigate these outcomes. Our study did not measure AMPA, a key metabolite of GLY. GLY exposure estimates might have been further improved by AMPA measurements but no well-established and reliable analytical method is known that can be used to measure AMPA in the urine matrix. Thus, we were unable to determine whether AMPA is an independent additive risk factor in pregnancy outcomes.

## Conclusions

Glyphosate is found at quantifiable levels in over 90% of pregnancies in the Indiana cohort. No drinking water samples had quantifiable GLY levels; thus, the source of exposure is most likely not drinking water. Women with higher caffeine intake or living in rural areas were found to have higher urine GLY levels. Higher GLY levels were significantly associated with shorter gestational lengths. The high frequency of GLY exposure (> 90%) found in this study, combined with supportive evidence of shortened pregnancy length, mandates further research. This study is significant because to our best knowledge, this is the first US study designed specifically to measure prenatal GLY exposure in pregnant women to determine its association with adverse fetal developmental risk. Additionally, the novel data on urinary GLY exposure in pregnant women and potential exposure pathways provide baseline information that is needed for designing future reproductive toxicological and epidemiological studies.

## Abbreviations

AMPA: α-amino-3-hydroxy-5-methyl-4-isoxazolepropionic acid
BMI: Body Mass Index
CFIA: Canadian Food Inspection Agency
CV: Coefficient of Variance
EFSA: European Food Safety Authority
ESI: Electrospray Ionization
FDA: Food and Drugs Administration
GC-MS: Gas Chromatography-Mass Spectrophotometery
GLY: Glyphosate
HPLC/MS: High Performance Liquid Chromatography-tandem Mass Spectrometry
IQR: Inter-Quartile Range
LC/MS/MS: Liquid Chromatography-tandem Mass Spectrometry
LOD: Limit of Detection
LOQ: Limit of Quantification
NA: Not applicable
NR: Not reported
SD: Standard Deviation
